# Social inequalities in the surrounding areas of food deserts and food swamps in a Brazilian metropolis

**DOI:** 10.1186/s12939-021-01501-7

**Published:** 2021-07-21

**Authors:** Olivia Souza Honório, Milene Cristine Pessoa, Lucia Helena Almeida Gratão, Luana Lara Rocha, Inês Rugani Ribeiro de Castro, Daniela Silva Canella, Paula Martins Horta, Larissa Loures Mendes

**Affiliations:** 1 Departamento de Nutrição, Universidade Federal de Minas Gerais, Avenida Professor Alfredo Balena,190, Santa Efigênia, Belo Horizonte, 30130-090 Brazil; 2 Faculdade de Medicina, Universidade Federal de Minas Gerais, Avenida Professor Alfredo Balena,190, Santa Efigênia, Belo Horizonte, 30130-090 Brazil; 3 Universidade do Estado do Rio de Janeiro, Instituto de Nutrição, Rua São Francisco Xavier,524,Maracanã, Rio de Janeiro, 20550-170 Brazil

**Keywords:** Food Desert, Food swamp, Social inequalities, Public policy, Food environment

## Abstract

**Background:**

Food deserts are neighborhoods with little or no access to healthy food, whereas food swamps are neighborhoods where unhealthy food options prevail over healthy ones. The main aims of the current study are to feature and compare the neighborhoods of food deserts and food swamps based on social inequality.

**Methods:**

Ecological study carried out in Belo Horizonte City, Minas Gerais State, Brazil. Information about commercial food establishments derived from two different databases. It was measured by secondary governmental databases, which were virtually conferred in the present study. Census tracts were considered as analysis units and classified as food deserts and food swamps, based on the Brazilian methodology. Take into consideration the density of establishments classified as selling fresh or minimally-processed food, mixed establishments, and establishments selling ultra-processed food. The Brazilian methodology evaluates food deserts by the density of healthy establishments (establishments classified as mostly selling fresh or minimally-processed food and mixed establishments) per 10 thousand inhabitants. And the metric to evaluate food swamps considers the density of unhealthy establishments (establishments mostly selling ultra-processed food) per 10 thousand inhabitants. Information about social inequalities comprised aspects such as income, population count, number of households, number of literate individuals, race, water and energy supply, and garbage collection. The Health Vulnerability Index (HVI) was used as a synthetic social vulnerability indicator.

**Results:**

Neighborhoods of food deserts presented worse essential service availability, lower income per capita, and smaller mean number of literate individuals. Census tracts classified as food swamps presented better socio-demographic conditions than those areas food deserts. Neighborhoods simultaneously classified as food deserts and food swamps presented lower income per capita and were more often observed in census sectors presenting medium and high HVI.

**Conclusion:**

The food environment in Belo Horizonte was featured by the strong presence of food deserts and food swamps. However, the potential influence of these areas on food intake has changed depending on social inequalities.

**Supplementary Information:**

The online version contains supplementary material available at 10.1186/s12939-021-01501-7.

## Background

Access to food comprises several dimensions, such as food availability, which lies in the presence of food in specific geographic areas [1]. Metaphors such as “food deserts” and “food swamps” can be used to describe this access, as well [2]. Although different concepts and methodologies can be used to describe these metaphors. Overall, food deserts are socially vulnerable neighborhoods with little or no access to healthy food, whereas food swamps are neighborhoods where unhealthy food options prevail over healthy ones [3].

Several studies have been conducted to help better understand the association of food deserts and food swamps with neighborhood and individual aspects. Neighborhoods with limited access to healthy food also had limited access to other services such as transportation, health services, and parks [4]. Concerning individual aspects, living in these neighborhoods was associated with an increased prevalence of chronic non-communicable diseases [5–7]. Moreover, individuals’ health conditions are also linked to social inequality, which encompasses aspects like income, schooling, professional career, sex, ethnicity, and neighborhood [8].

Furthermore, few studies evaluate the food deserts and food swamps in middle- and low-income countries, and in Brazil, the studies are focused on the assessment of food deserts [9, 10]. The studies of the topic in middle- and low-income countries are fundamental because the food environment in these places has many particularities [10, 11]. Moreover, in Brazil, data indicate that neighborhoods with greater inequity have worse access to health services [12], and there is also an increase in the population’s situation of food insecurity [13].

In this sense, the association between food deserts and food swamps as features of social inequality in developing countries remains poorly understood. Thus, the present study aimed to feature and compare the neighborhoods of food deserts and food swamps, based on social inequality.

## Methods

### Study design

An ecological study was carried out in Belo Horizonte City, capital of Minas Gerais State, Brazil. Belo Horizonte is the sixth-largest city in the country; it presents an estimated population of 2,375,151 inhabitants, a demographic density of 7167 inhabitants/km^2^, and Municipal Human Development Index (*Índice de Desenvolvimento Humano Municipal*’ - IDHM) of 0.810 [14].

Census tracts were the analysis units adopted in the current study. One hundred and six (106; 2.7%) of the 3936 census tracts observed in the city were excluded because they lacked the essential information to be analyzed in the current study.

### Study variables

#### Food retail information

Database comprising information collected in two secondary data sources, namely: (I) Superintendence for Tax Collection and Information of Minas Gerais State Finance Department (*Superintendência de Arrecadação e Informações Fiscais da Secretaria da Fazenda do Estado de Minas Gerais*) and (II) Assistant Inspection Department (*Secretaria Municipal Adjunta de Fiscalização*). Databases comprised information about the address and the National Classification of Economic Activities (*Classificação Nacional de Atividades Econômicas* - CNAE) of 13 food retailer categories registered in Belo Horizonte in 2015 [15]. CNAE is an instrument used nationwide to standardize activity codes for economic situation and criteria used by several Tax Administration institutions in the country [16].

Commercial establishments whose information was matching in the two data sources were included in the final database, based on the identification of their corporate names, which was a common variable between them. Images from 2015, found through addresses registered in the Google Street View tool available at Google Maps application (https://www.google.com.br/maps), were used to check establishments with mismatched information [15]. This application shows a panoramic view of the streets; thus, establishments whose information did not match in the two secondary databases - which were considered as existing in the virtual checking process - were also included in the final database [15]. CNAE available in the database was corrected based on the updated CNAE record, whenever it was at odds with what was identified during the virtual checking [15]. Commercial establishments presenting mismatching in the databases and that were not identified in Google Street View were excluded from the study [15]. Given the need of conducting virtual checking, establishments classified as mobile food services (CNAE 5612100) were also excluded from the sample, since they do not operate in a fixed place or at all times and days [15].

Also, Public Establishments for Food Security, services focused on supplying, distributing, and selling meals or food [17] in Belo Horizonte City were included in the final database, which comprised 15,455 food retailers [15].

Food retailers were categorized as (I) establishments mostly selling fresh or minimally-processed food, mainly fresh food (50% or higher), (II) mixed establishments (the ones selling fresh or minimally-processed and ultra-processed food), and (III) establishments mostly selling ultra-processed food (50% or higher) [10]. It was done based on the classification suggested by CAISAN (Table 1).

**Table 1:** Food retailer classification based on CAISAN

Classification	Food retailers
Establishments mostly selling fresh or minimally-processed food	Public Establishments for Food Security, Fresh product store, Butcher shop, Fish market
Mixed establishments	Restaurants, Bakery, Minimarkets, Grocery stores, Supermarkets, Dairy products
Establishments mostly selling ultra-processed food	Pubs, Snack bars, Candy shops

Source: Adapted from CAISAN,2018 [13]

#### Social inequality variables

Information on variables such as income, population count, number of households, number of literate individuals, race, and essential services (water supply, garbage collection, and electricity) were collected at the 2010 IBGE Census database [18]. Mean monthly income per capita was calculated by dividing the total income of the census tracts by the total population living in the census tracts; it was categorized based on distribution quartiles, namely: 1st quartile: from $ 46.83 to $ 158.61; 2nd quartile: from $ 158.62 to $ 250.24; 3rd quartile: from $ 250.25 to $ 562.85; and 4th quartile: values ​​higher than $ 562.85, according to the dollar exchange rate on December 30th, 2010.

Variable “race” was grouped into three categories: white, mixed, and Asian descendant/indigenous [19]; the mean number of individuals in each category was calculated for this variable. Individuals in the age group 5 years or older who could read or write a simple note were considered literate [18]. The mean number of literate individuals in the census sectors was calculated to describe literacy in them.

The number of households served with water and electricity supply, and garbage collection in the census tracts, as well as the Health Vulnerability Index (HVI), were used to describe the census tracts. HVI is a synthetic indicator used to assess the degree of vulnerability of census tracts [20]. This index evaluates variables associated with basic sanitation and socioeconomic status [20].

Aspects associated with basic sanitation comprised the rate of households with inadequate or lacking water supply, with inadequate or lacking garbage collection, and with inadequate or lacking sanitary sewage.

Overall, the herein evaluated socioeconomic features comprised the mean number of residents per household, the rate of illiterate individuals and households whose income per capita was lower than half minimum wage, mean monthly income, and rate of mixed-race or indigenous individuals [20]. According to HVI, census tracts can be classified in four groups: low HVI, medium HVI, high HVI, and very high risk [20]. Variable HVI was herein addressed based on three classifications: low risk, medium risk, and high-risk. The high-risk category has grouped high and very high-risk categories [20].

#### Food deserts and food swamps

The Brazilian methodology to evaluate food deserts was proposed by the Inter-Ministerial Chamber of Food and Nutritional Security (acronym in Portuguese CAISAN). The proposed calculation of food swamps considers a calculation proposed by CAISAN, in the Technical Study on Mapping Food Deserts in Brazil. CAISAN is a governmental body, whose function is to elaborate and monitor public policies of food and nutritional security.

The proposal by CAISAN [10] was applied to identify food deserts, by using census tracts as analysis units. Based on this methodology, food deserts were identified by calculating the density of establishments classified as mostly selling fresh or minimally processed food and of mixed establishments per 10,000 inhabitants [10]. Food deserts were considered census tracts whose density of fresh or minimally-processed food and mixed establishments was below the 25th percentile of the distribution [10] in all census tracts in Belo Horizonte City.

The Brazilian methodology also includes calculating the density of establishments mostly selling ultra-processed food, which corresponds to the total number of these establishments in the census tracts divided by 10,000 inhabitants [10]. Food swamps were identified by calculating the density of establishments mostly selling ultra-processed food per 10,000 inhabitants; the adopted criterion lied on the census tracts whose density of establishments selling ultra-processed food was above the 25th percentile of their distribution in all census tracts in Belo Horizonte City [21].

It is worth clarifying that some census tracts can be simultaneously classified as food deserts and food swamps. These census tracts have limited geographic access to establishments mainly focused on selling fresh or minimally-processed food and to mixed establishments. On the other hand, they have easy geographic access to establishments mostly selling ultra-processed food.

### Data analysis

Descriptive analysis was applied to frequency distribution, measures of central tendency, and variables’ dispersion (sociodemographic and essential services), based on the classification of food deserts and food swamps. Also, two statistical tests, chi-square and Student’s t-test were applied to compare the differences between proportions and means, respectively. The significance level adopted was *p* < 0.05. The analyses were performed in the QGIS 2.14.9 and SPSS 19.0 software.

## Results

In total, 37.80% (*n* = 1444) of census tracts were classified as food deserts (31.20% of the population lived in these places), whereas 58.50% (*n* = 2240) of them were classified as food swamps (64.00% of the population lived in these places). On the other hand, 12.74% of census tracts were simultaneously classified as food deserts and food swamps. It is worth emphasizing that 83.53% of the population lived in census tracts classified as food deserts and/or food swamps (Table 2).

**Table 2:** Featuring census tracts’ population based on their classification as food deserts and food swamps

Census tract features	FOOD DESERT		FOOD SWAMPS		Food deserts and food swamps at the same time^**a**^ (***n*** = 487; 12.74%)
	Yes(n = 1444; 37.80%)	No(***n*** = 2386; 62.2%)	Yes(***n*** = 2240; 58.50%)	No(***n*** = 1590; 41.50%)	
	Mean ± SD/ %	Mean ± SD/ %	Mean ± SD/ %	Mean ± SD/ %	Mean ± SD/ %
**Income per capita (R$)**^b*+^	631.44 ± 714.10	816.93 ± 857.64	787.85 ± 783.27	689.44 ± 846.43	595.32 ± 655.3^≠^
**Total Population**^b*+^	511.88 ± 310.10	683.76 ± 290.95	682.43 ± 294.08	529.54 ± 309.15	590.47 ± 296.74
**Number of households**^b*+^	162.53 ± 99.57	220.84 ± 89.47	220.28 ± 90.73	168.68 ± 98.92	186.65 ± 93.90^≠^
**Number literate individuals**^b*+^	457.97 ± 277.18	625.14 ± 261.04	623.97 ± 264.51	474.97 ± 276.21	530.99 ± 265.99
***Race***^***c***^					
White*^+^	43.16	48.38	47.89	44.69	42.18^≠^
Mixed*^+^	55.52	50.45	50.93	54.05	56.55^≠^
Asian descendant/Indigenous*^+^	1.30	1.16	1.18	1.27	1.27^≠^
***Health Vulnerability Index***^***d***^					
Low*^+^	27.70	39.00	37.10	31.30	24.40^≠^
Medium*^+^	31.60	42.10	42.80	31.50	38.60
High*^+^	40.70	18.90	20.10	37.20	37.00^≠^

Note: * Statistical difference between food deserts and no- food deserts; ^+^ Statistical difference between food swamps and no- food swamps; ^≠^ Statistical difference; ^a^ Comparison as the sectors that are neither deserts nor swamps (S1); ^b^ *p*-value calculated by the t-test, ^c^ Result expressed in percentage of individuals and *p*-value calculated by the chi-square; ^d^ Result expressed in percentage of census tract and p-value calculated by the chi-square; p-value < 0,05

Table 2 describes the sociodemographic features of the population living in census tracts classified as food deserts and food swamps. Census tracts classified as food deserts recorded lower mean income per capita (631.44 ± 714.10; *p* < 0.0001) than the other census tracts classified as non-food deserts, food swamps, or non-food swamps. Also, food deserts recorded lower mean for variables such as total population, number of households, and number of literate individuals than that recorded for the other census tracts (Table 2).

Concerning variable “race”, the mixed-race category was more often observed in census tracts classified as food deserts (55.52%; *p* < 0.0001) and in the ones classified as food swamps (50.93%; p < 0.0001) (Table 2). On the other hand, census tracts classified as food deserts were more often observed in neighborhoods with high HVI (40.70%; p < 0.0001), whereas census tracts classified as food swamps were more often observed in neighborhoods with medium (42.80%; p < 0.0001) and low HVI (37.10%).

Census tracts simultaneously classified as food deserts and food swamps presented lower mean income per capita (595.32 ± 655.11; p < 0.0001). These census tracts were more often observed in neighborhoods with medium and high HVI.

Table 3 describes the supply of essential services for households located in census tracts classified as food deserts and food swamps. The mean number of households provided with essential services in census tracts classified as food deserts was smaller than that of households provided with essential services in census tracts classified as food swamps. Census tracts classified as food deserts and the ones classified as non-food swamps presented a similar mean number of households provided with essential services. Census tracts classified as food swamps presented a higher mean number of households supplied with all essential services than that recorded for other census tract classifications.

**Table 3:** Mean number of places according to essential service types available in the census tract

Census tract features	FOOD DESERT		FOOD SWAMPS		Food deserts and food swamps at the same time(n = 487)^**a**^
	Yes(n = 1444)	No(n = 2386)	Yes(n = 2240)	No (n = 1590)	
	Mean ± SD	Mean ± SD	Mean ± SD	Mean ± SD	Mean ± SD
***Water supply***					
General network*^+^	161.76 ± 99.45	220.11 ± 89.31	219.46 ± 90.61	168.03 ± 98.81	185.87 ± 93.82 ^≠^
**Other supply forms**	0.22 ± 1.39	0.32 ± 2.13	0.32 ± 2.13	0.22 ± 1.47	0.18 ± 0.82
***Garbage collection***					
Collected garbage*^+^	160.95 ± 99.85	219.93 ± 89.46	219.32 ± 90.82	167.22 ± 99.11	185.26 ± 94.33 ^≠^
Garbage collected by cleaning service*^+^	156.12 ± 100.48	217.45 ± 90.53	216.29 ± 92.10	163.38 ± 100.03	179.38 ± 95.85 ^≠^
Garbage collected in cleaning service bucket*	4.83 ± 22.18	2.48 ± 17.51	3.03 ± 19.73	3.84 ± 19.02	5.88 ± 26.72
**Others**	1.25 ± 9.88	0.72 ± 7.98	0.76 ± 8.63	1.14 ± 8.91	1.19 ± 10.50
***Electric power***					
Permanent private places with electricity*^+^	162.23 ± 99.53	220.62 ± 89.39	220.06 ± 90.65	168.38 ± 98.88	186.45 ± 93.89 ^≠^
Electricity from distribution company*^+^	161.06 ± 99.43	220.08 ± 89.47	219.56 ± 90.67	167.20 ± 98.88	185.94 ± 93.72
**Permanent private places without electricity**	0.07 ± 0.35	0.08 ± 0.34	0.07 ± 0.33	0.08 ± 0.36	0.07 ± 0.31

Note:; * Statistical difference between food deserts and no- food deserts; ^+^ Statistical difference between food swamps and no- food swamps; ^≠^ Statistical difference ^a^ Comparison as the sectors that are neither deserts nor swamps (S1);p-value calculated by the t-test; p-value < 0,05

## Discussion

Most of the investigated population was exposed to food environments that did not favor healthy eating practices, since more than 80% of them lived in neighborhoods classified as food deserts and/or food swamps. Moreover, census tracts classified as food deserts presented worse sociodemographic conditions and access to essential services than neighborhoods classified as non-food deserts. On the other hand, neighborhoods classified as food swamps have shown better sociodemographic conditions and access to essential services than neighborhoods classified as non-food swamps.

Results recorded for food deserts were similar to those observed in studies carried out in developed countries, where food deserts were observed in regions subjected to greater sociodemographic vulnerability [4, 5, 22, 23]. However, it is necessary to be careful at the time to make comparisons, since the food environment in these countries is different from the Brazilian reality. Also, food deserts were classified based on a different methodology. The main difference lies in the fact that most international methodologies only take into consideration the geographic access to supermarkets at the time to classify the investigated neighborhoods [4, 22, 23], whereas the current study took into consideration a large number of food retailers.

Results of studies carried out in low-income countries and emerging economies remain incipient and controversial when it comes to assessing food deserts. A study carried out in three communities with different socioeconomic levels in Mexico did not find food deserts and all evaluated neighborhoods presented availability of healthy food stores [11]. However, lower-income communities had limited economic access to healthy food compared to the wealthier ones [11, 24]. Also, some studies have indicated that poorer neighborhoods had worse economic access to healthy food [9, 25]. However, a study conducted in Brazil did not show an association between food deserts and neighborhood income [9].

Concerning ethnic aspects, studies carried out in the United States have shown an association between race and food deserts. Black and Latino populations were more susceptible to live near food deserts; the main explanation for this association lies in spatial segregation resulting from the lower-income of these populations [22, 26]. The rate of mixed-race individuals in the current study was higher in neighborhoods classified as food deserts. This outcome reflects a Brazilian population feature since mixed-race individuals often have lower income and schooling than white ones [27].

This is the first study focused on investigating essential services’ supplying in food deserts and food swamps in Brazil, whose evaluation may represent a proxy for urban and health disparities [28]. Census tracts classified as food deserts presented worse availability of essential services in comparison to all others. This outcome has highlighted social inequality and the lack of actions taken by public authorities in these places.

Food swamps have gained prominence in comparison to food deserts since they show availability and excessive access to ultra-processed food; this feature is characteristic of food environments mostly associated with unhealthy food intake and, consequently, with the onset of Non-Communicable Diseases (NCDs) [11, 29, 30]. Studies available in the international literature reported food swamps in all regions, regardless of socioeconomic classification [23, 31–35].

Besides, studies conducted in Brazil have shown that social inequality and access to essential services can influence different aspects of populations’ lives. One study has found that the basic infrastructure of essential services can influence both income distribution and health perception in the investigated neighborhood [36]. Studies have also found an association between social inequality, exercising [37], access to and intake of food among Brazilians [38]. Socially disadvantaged individuals were the most underprivileged in these aspects [37, 38].

In addition, there is already evidence that access to essential services contributes to health outcomes; for example, limited access to water is associated with the scenario of malnutrition [39, 40], and increased consumption of unhealthy food [40]. Other consequences of low access to water are difficulty in food preparation and reduced food diversity [41]. In addition, in a study conducted in a middle-income country, an association was found between limited access to water and food with higher susceptibility to chronic diseases and diseases communicable by these families [42].

Finally, investigating the neighborhoods of food deserts and food swamps can be important tools to guide the development and implementation of food and nutrition public policies. By identifying the neighborhoods of food deserts, it is possible to think of places that require policies to increase access to healthy food, such as public establishments for food security. And in the neighborhoods of food swamps, is necessary structural actions that reduce the exposure and access of individuals to unhealthy foods, such policies related to the taxation of sugary drinks and regulation of food advertising.

The current study has some limitations, such as the fact that it was not possible to evaluate the informal food trade because the study used secondary databases. The second limitation is associated with the temporality of the analyzed data since information about sociodemographic features and access to essential services derived from the 2010 Census. It is worth emphasizing that these data were used because it was the last census carried out in the country. The third limitation refers to the methodology used to classify food deserts and food swamps, which was recently developed.

However, the strengths of the current study lie in using population-based data and in addressing sociodemographic aspects of all individuals living in Belo Horizonte City. Furthermore, different types of ready-to-eat food retailers (snack bars, pastry shops, and bars) were taken into consideration at the time to calculate food swamps in the current study, whereas several international studies only take into consideration fast-food restaurants. The methodology used to evaluate food deserts respected the peculiarities of the local food environment. Finally, the current study adopted a different approach, which associated community food environment features with access to essential services.

## Conclusion

The worst socio-demographic characteristics were found in neighborhoods classified as food deserts. This study has shown that neighborhoods classified as food deserts not only require interventions focused on changing their food environment, but they also need to improve their social environmental conditions.

Furthermore, neighborhoods classified as food deserts and food swamps simultaneously should receive special attention in the development of public nutrition policies. Since, in terms of exposure to the food environment, residents in these neighborhoods are exposed to a food environment that favors unhealthy food choices.

Finally, metrics for investigating food deserts and food swamps in low and middle-income countries still need to be refined. These advances will facilitate further investigations into the association of food deserts and food swamps with health outcomes.

## Supplementary Information

**Additional file 1 S1**: Descriptive census tracts that are neither food deserts nor food swamps.

## Data Availability

The datasets used and/or analysed during the current study are available from the corresponding author on reasonable request.

## Abbreviations

CAISAN: Inter-Ministerial Chamber of Food and Nutritional Security/ Camara Interministerial de Segurança Alimentar e Nutricional
CNAE: National Classification of Economic Activities
HVI: Health Vulnerability Index
IDHM: Municipal Human Development Index
NCDs: Non-Communicable Diseases
