# Supplementary material for: Social inequalities in the surrounding areas of food deserts and food swamps in a Brazilian metropolis
Source: Int J Equity Health. 2021 Jul 21;20:168. doi: 10.1186/s12939-021-01501-7 (PMC8293554; doi:10.1186/s12939-021-01501-7)
Supplement: Supplementary file 1 — Additional file 1 S1: Descriptive census tracts that are neither food deserts nor food swamps. [file 12939_2021_1501_MOESM1_ESM.docx]

| ***S1****: Descritivo setores censitários que não são desertos e nem pântanos alimentares* | |
| --- | --- |
|  | *Mean ±SD/ %* |
| **Income per capita (R$)** | 749.33*±*981.21 |
| **Total Population** | 616.72*±*288.07 |
| **Number of households** | 196.52*±*90.19 |
| **Number literate individuals** | 556.85*±*256.61 |
| ***Race**** | |
| White | 45.73 |
| Mixed | 53.09 |
| Asian descendant/Indigenous | 1.18 |
| ***Health Vulnerability Index **** | |
| Low | 34.3 |
| Medium | 36.8 |
| High | 28.9 |
| ***Water supply*** | |
| General network | 196.06*±*90.01 |
| Other supply forms | 0.21*±*1.25 |
| ***Garbage collection*** | |
| Collected garbage | 195.39 *±*90.24 |
| Garbage collected by cleaning service | 192.24*±*91.73 |
| Garbage collected in cleaning service bucket | 3.15*±*18.32 |
| Others | 0.92*±*7.85 |
| ***Electric power*** | |
| Permanent private places with electricity | 196.33*±*90.14 |
| Electricity from distribution company | 195.63*±*90.20 |
| Permanent private places without electricity | 0.08*±*0.36 |

Note: *percentage (%)
